# Association of Chronic Obstructive Pulmonary Disease With Increased Confusion or Memory Loss and Functional Limitations Among Adults in 21 States, 2011 Behavioral Risk Factor Surveillance System

**DOI:** 10.5888/pcd13.150428

**Published:** 2016-01-07

**Authors:** Kurt J. Greenlund, Yong Liu, Angela J. Deokar, Anne G. Wheaton, Janet B. Croft

**Affiliations:** Author Affiliations: Yong Liu, Angela J. Deokar, Anne G. Wheaton, Janet B. Croft, Centers for Disease Control and Prevention, Division of Population Health, National Center for Chronic Disease Prevention and Health Promotion, Atlanta, Georgia.

## Abstract

**Introduction:**

Chronic obstructive pulmonary disease (COPD) is associated with cognitive impairment, but consequences of this association on a person’s functional limitations are unclear. We examined the association between COPD and increased confusion and memory loss (ICML) and functional limitations among adults with COPD.

**Methods:**

We studied adults aged 45 years or older in 21 states who participated in the 2011 Behavioral Risk Factor Surveillance System (n = 102,739). Presence of COPD was based on self-reported physician diagnosis. ICML was based on self-report that confusion or memory loss occurred more often or worsened during the prior year. ICML-associated difficulties were defined as giving up household chores and former activities, decreased ability to work or engage in social activities, or needing help from family or friends during the prior year due to ICML. General limitations were defined as needing special equipment as a result of a health condition, having had activity limitations for 2 weeks or more in the prior month, or being unable to work. Multivariable models were adjusted for demographics, health behaviors or conditions, and frequent mental distress.

**Results:**

COPD was reported by 9.3% of adults. ICML was greater among those with COPD than among those without COPD (25.8% vs 11%; adjusted prevalence ratio [aPR], 1.48; 95% confidence interval [CI], 1.32%–1.66%). People with COPD, either with or without ICML, were more likely than those without COPD to report general functional limitations. Among people reporting ICML, those with COPD were more likely to report interference with work or social activities than those without COPD (aPR, 1.17; 95% CI, 1.01%–1.36%).

**Conclusion:**

Functional limitations were greater among those with COPD than among those without, and ICML may further affect these limitations. Results from our study can inform future studies of self- management and functional limitations for people with COPD.

## Introduction

Cognitive impairment among people with chronic obstructive pulmonary disease (COPD) has been observed in community populations (1–4). Details of the association between cognitive impairment and COPD are emerging; however, its consequences for people living with COPD are unclear. A systematic review concluded that future research on the effects of cognitive impairment on people living with COPD should include its impact on daily living and self-care (5). At least 1 study observed that adults with COPD had more functional limitations in activities of daily living and self-care than adults without COPD (6). However, the impact of cognitive impairment on activities of daily living and self-care among adults with COPD has received little attention and requires assessment. 

Cognitive health issues may range from mild impairment to some type of dementia, including Alzheimer’s disease. People with COPD may have overall cognitive impairment or impairment in specific cognitive domains that affect information processing, attention, concentration, memory, executive functioning, and self-control (5). Memory problems are a common early sign of cognitive impairment (7), which may begin many years before it is clinically diagnosed. Therefore, self-reported memory problems have generated interest as a marker of cognitive decline. People who complain about memory loss appear to be at greater risk of developing mild cognitive impairment or dementia (8–11). However, some studies suggest that complaints about memory loss may be related to psychological distress and not necessarily to cognitive impairment (8,10,12).

Cognitive impairment in conjunction with COPD may be related to hypoxia or hypercapnia (5,13) or to acute exacerbations of COPD (14). In addition, cigarette smoking, a major cause of COPD, is associated with decreased cognitive function and increased cognitive decline (4,15–17); the 2011 Behavioral Risk Factor Surveillance System (BRFSS) showed that almost 40% of adults with COPD that year were smokers (18). Furthermore, COPD frequently co-occurs with other chronic conditions (19–21), many of which also can involve cognitive impairment, such as heart and vascular diseases (22,23), diabetes (24), and stroke (23).

The 2011 BRFSS collected self-reported information on both physician-diagnosed COPD (16) and on confusion or memory loss (25,26) among participants in 21 states. This large sample of more than 100,000 respondents is probably reflective of the heterogeneous population that physicians encounter in general practice. We conducted cross-sectional analyses to examine 1) the association between COPD and increased confusion or memory loss (ICML), adjusting for potential confounders, and 2) differences in functional limitations by ICML and COPD status, Results of our analyses can inform self-management and community-based programs about the cognitive and functional needs of people with COPD.

## Methods

BRFSS is a state-based telephone survey of adults aged 18 years or older, which is conducted by state health departments with assistance from the Centers for Disease Control and Prevention (CDC). BRFSS samples people with landline and people with cellular telephones to obtain representative samples of the noninstitutionalized adult population. Data are weighted to state population estimates using iterative proportional fitting or “raking.” Details on BRFSS design, methodology, sampling procedures, reliability, and validity are available (www.cdc.gov/brfss). The survey includes core questions asked in all states and optional modules on specific topics, which states select based on their programmatic needs. Optional modules may include the full state sample or split samples to allow states to obtain information on more public health topics of interest to the state programs. In 2011, COPD status was part of the core BRFSS survey. An optional module asked 138,874 people in 21 states about increased confusion or memory loss (ICML) and associated functional difficulties. The 21 states were Arkansas, California, Florida, Hawaii, Illinois, Iowa, Louisiana, Maryland, Michigan, Nebraska, New Hampshire, New York, North Carolina, Oklahoma, South Carolina, Tennessee, Texas, Utah, Washington, West Virginia, and Wisconsin. The median response rate was 53.4% using response rate standards of the American Association of Public Opinion Research (http://www.aapor.org/AAPORKentico/Communications/AAPOR-Journals/Standard-Definitions.aspx). BRFSS procedures were reviewed by the CDC Human Research Protections Office and determined to be exempt research. All analyses were performed using data from the BRFSS public use files.

We determined COPD status from responses to the BRFSS question, “Have you ever been told by a doctor or health professional that you have COPD, emphysema, or chronic bronchitis?” Increased confusion or memory loss was determined from responses to the question, “During the past 12 months, have you experienced confusion or memory loss that is happening more often or is getting worse?” We examined both ICML-associated functional difficulties (questions asked only of those reporting ICML) and general physical limitations (questions asked of all adults regardless of ICML status). BRFSS assessed ICML-associated difficulties by asking respondents who reported ICML how often in the previous year 1) they gave up household activities or chores they formerly engaged in because of ICML or 2) how often in the previous year ICML interfered with their ability to work, volunteer, or engage in social activities. We grouped responses for these 2 questions as “always,” “usually,” or “sometimes” versus “rarely” or “never.” Respondents also noted in which areas they needed most help because of ICML (safety, transportation, household activities, personal care, some other area, or no areas). We determined whether a respondent needed help in any category of help compared with needing no help. Additionally, respondents noted if they received help from family or friends in the previous 30 days because of ICML; we categorized responses as “always,” “usually,” or “sometimes” versus “rarely” or “never.”). We examined 3 general areas of physical or mental limitations. First we determined whether a respondent needed special equipment because of a health condition. Second we determined whether a respondent had 2 or more weeks of limited physical activity in the prior month; such determination was based on responses to questions asking whether respondents had any days when physical health was not good, had days when mental health was not good, and the number of days that poor physical or mental health kept them from their usual activities (eg, self-care, work, recreation). Third, we determined respondents’ employment status from reports of “unable to work.” Specific health reasons for these general limitations were not ascertained. 

Demographic variables used in our analyses were age, sex, race/ethnicity (non-Hispanic white, non-Hispanic black, Hispanic, all others), education (less than high school, high school or equivalent, more than high school), number of adults in the household (1, ≥2), health care insurance coverage, and state of residence. Health behaviors used were smoking status, obesity status, and physical inactivity. Current smokers were those who smoked at least 100 cigarettes in their lifetime and currently smoked every day or some days. Former smokers were those who smoked at least 100 cigarettes in their lifetime but did not currently smoke. Never smokers were those who had never smoked at least 100 cigarettes in their lifetime. Body mass index (BMI), calculated from self-reported height and weight, was categorized as underweight (BMI <18.5 kg/m^2^), normal weight (BMI 18.5–24.9 kg/m^2^), overweight (BMI 25.0–29.9 kg/m^2^), and obese (BMI ≥30 kg/m^2^). Respondents were asked if they engaged in any leisure time physical activity in the prior month (yes/no). Chronic disease risk factors and conditions were determined by asking, “Have you ever been told by a doctor or health professional that you have . . . high blood pressure, high cholesterol, heart disease (myocardial infarction or coronary heart disease), stroke, diabetes, or current asthma?” Finally, frequent mental distress (defined as psychological distress experienced for 14 or more days in the prior month) was assessed with the question, “Now thinking about your mental health, which includes stress, depression, and problems with emotions, for how many days during the past 30 days was your mental health not good?” (27). For our purposes, frequent mental distress captures more than clinically diagnosed depression and reflects a wider range of psychological distress.

We combined data for the 21 states and restricted analyses to respondents aged 45 years or older who were not missing data for COPD status (N = 105,332: 10,476 with COPD and 94,856 without COPD), because the sample sizes for those with COPD younger than 45 were too small for reliable comparisons. We used appropriate population weights for full and split samples for the ICML module. SAS (SAS Institute) and SAS-callable SUDAAN (Research Triangle Institute) were used to account for the complex sampling design and for variance estimation. We first examined differences in demographic and health characteristics by COPD status. We then examined differences in ICML by COPD status. Finally, we compared functional limitations by ICML and COPD status. Adjusted prevalence ratios were calculated by using 3 models. Model 1 was adjusted for sex, race/ethnicity, education, number of adults in the household, health care coverage, state of residence, and age (as a continuous variable). Model 2 was adjusted for model 1 covariables plus health behaviors and risk factors (smoking, obesity status, physical inactivity, high blood pressure, high cholesterol) and presence of at least 1 other chronic condition (heart disease, stroke, diabetes, current asthma). Model 3 was adjusted for models 1 and 2 plus frequent mental distress. For descriptive analyses, sample sizes were allowed to vary. Multivariable analyses were restricted to respondents with no missing data for all covariables so we could compare prevalence ratios across models for the relevant outcome. Significance was determined as *P* < .05. We checked relative standard errors to assess stability of estimates. 

## Results

Among people aged 45 years or older in the 21 states studied, the weighted percentage with COPD was 9.3% (95% confidence interval [CI], 8.9%–9.7%). Compared with those without COPD, a higher percentage of people with COPD were aged 65 to 84 years, were women, were white, had a high school education or less, were current smokers, were physically inactive, were obese, had other chronic disease risk factors and conditions, and had frequent mental distress (Table 1). People with COPD were less likely to report Hispanic ethnicity.

**Table 1:** Demographic and Health Characteristics of Adults Aged 45 Years or Older in 21 States^a^, by COPD Status, 2011 Behavioral Risk Factor Surveillance System

Characteristic	With COPD (N = 10,476)		Without COPD (N = 94,856)	
	n	% (95% CI)^b^	n	% (95% CI)^b^
**Age, y**				
45–54	1,670	25.3 (23.2–27.6)	22,522	37.8 (37.0–38.5)
55–64	3,000	29.7 (27.7–31.8)	28,583	29.2 (28.6–29.9)
65–75	3,173	24.7 (22.9–26.7)	23,485	17.7 (17.2–18.2)
75–84	2,116	16.5 (15.3–17.9)	15,335	11.8 (11.4–12.2)
≥85	517	3.7 (3.1–4.3)	4,931	3.4 (3.2–3.6)
**Women**	7,043	59.2 (56.8–61.5)	59,021	52.5 (51.7–53.2)
**Race/ ethnicity**				
White non-Hispanic	8,485	74.8 (72.4–77.1)	75,095	69.2 (68.4–69.9)
Black non-Hispanic	901	9.5 (8.1–11.0)	8,985	10.2 (9.8–10.7)
Hispanic	260	7.7 (6.3–9.4)	3,683	12.8 (12.1–13.5)
Other	830	8.0 (6.5–9.8)	7,093	7.8 (7.3–8.3)
**Education**				
<High school	1,913	25.6 (23.4–27.9)	8,987	16.2 (15.6–17.0)
High school or equivalent	3,863	32.1 (30.2–34.1)	29,533	27.7 (27.1–28.4)
>High school	4,675	42.2 (40.1–44.4)	56,032	56.0 (55.3–56.8)
**Only adult in household**	5,004	32.3 (30.5–34.2)	35,047	23.2 (22.7–23.8)
**Have health insurance**	9,534	89.1 (87.4–90.6)	86,717	88.2 (87.6–88.8)
**Smoking status**				
Never smoker	2,435	21.5 (19.9–23.3)	51,046	52.6 (51.9–53.4)
Former smoker	4,693	44.9 (42.6–47.2)	31,392	32.7 (32.0–33.4)
Current smoker	3,311	33.6 (31.5–35.7)	11,937	14.6 (14.1–15.2)
**Physically inactive**	4663	45.0 (42.7–47.3)	25,674	27.6 (26.9–28.3)
**Body mass index (kg/m2)**				
Underweight (<18.5)	360	3.2 (2.6–4.0)	1,349	1.3 (1.2–1.5)
Normal weight (18.5–24.9)	2,865	27.1 (25.0–29.2)	29,450	30.6 (29.9–31.3)
Overweight (25.0–29.9)	3,181	31.7 (29.7–33.8)	34,263	38.0 (37.2–38.7)
Obese (≥30.0)	3,708	38.0 (35.7–40.3)	25,755	30.0 (29.3–30.8)
**Other chronic disease risk factors**				
High blood pressure	6,895	65.8 (63.6–67.8)	47,242	46.8 (46.0–47.5)
High cholesterol	6,135	65.8 (63.7–67.8)	43,488	48.6 (47.9–49.4)
**Chronic conditions**				
Heart disease	3,018	28.0 (26.0–30.0)	10,163	9.8 (9.4–10.3)
Stroke	1,291	11.1 (9.9–12.5)	4,774	4.3 (4.0–4.5)
Diabetes	2,681	25.5 (23.6–27.6)	14,980	15.9 (15.4–16.5)
Current asthma	3,600	36.7 (34.5–38.9)	5,242	5.9 (5.5–6.2)
Any chronic condition^c^	6,709	64.5 (62.3–66.6)	27,813	28.2 (27.5–28.9)
Frequent mental distress	2,426	26.9 (24.8–29.1)	8,254	10.5 (10.0–11.1)

Abbreviations: CI, confidence interval; COPD, chronic obstructive pulmonary disease.

a Arkansas, California, Florida, Hawaii, Illinois, Iowa, Louisiana, Maryland, Michigan, Nebraska, New Hampshire, New York, North Carolina, Oklahoma, South Carolina, Tennessee, Texas, Utah, Washington, West Virginia, and Wisconsin.

b Percentages are weighted to state population estimates. Totals may vary because of missing responses.

c Heart disease (heart attack, coronary heart disease), stroke, diabetes, current asthma.

Overall, 12.4% (95% CI, 11.9%–12.9%) of people reported ICML during the previous year, and ICML was greater among people with COPD (25.8%; 95% CI, 23.9-27.8) than among those without COPD (11.0%; 95% CI, 10.5–11.6). Overall, the percentage reporting ICML was higher among those reporting stroke (29.7%; 95% CI, 26.9–32.7) than it was among those with COPD (25.8%), but was lower for those reporting current asthma (22.3%; 95% CI, 20.1–24.6), heart disease (21.4%; 95% CI, 19.7–23.2) or diabetes (17.2%; 95% CI, 15.9–18.6).

Within each demographic, health behavior, or health condition group, the percentage with ICML was greater among those with COPD than without COPD (Table 2). The only exception was among underweight people, for whom the percentage of ICML did not differ by COPD status because of large confidence intervals. Interestingly, among those with COPD, the percentage with ICML declined with age, whereas ICML was slightly higher among those in the two oldest age groups of those without COPD. Among both those with and those without COPD, prevalence of ICML tended to be higher among Hispanics than whites, lower with more attained education, higher among current smokers than among former or never smokers, and higher among those with chronic conditions or frequent mental distress than among those without. Prevalence of ICML increased with body weight both among people with COPD and among those without except among underweight people without COPD. 

**Table 2:** Percentage of Adults Aged 45 Years or Older Reporting ICML, by Demographic and Health Characteristics, Among People With and People Without COPD, 21 States^a^, 2011 Behavioral Risk Factor Surveillance System

Characteristic	With COPD		Without COPD	
	N	% (95% CI)^b^	N	% (95% CI)^b^
Total	9,512	25.8 (23.9-27.8)	87,352	11.0 (10.5–11.6)
**Age, y**				
45–54	1,510	32.5 (27.7–37.8)	20,929	10.7 (9.7–11.8)
55–64	2,762	27.0 (23.5–30.9)	26,662	10.5 (9.7–11.5)
65–75	2,894	21.4 (18.2–25.0)	21,662	10.5 (9.6–11.4)
75–84	1,890	20.7 (17.6–24.2)	13,796	13.1 (11.9–14.4)
≥85	456	23.2 (17.2–30.4)	4,303	14.4 (12.4–16.7)
**Sex**				
Male	3,104	26.0 (22.6–29.7)	32,984	10.9 (10.1–11.8)
Female	6,408	25.7 (23.4–28.0)	54,368	11.1 (10.4–11.8)
**Race/ethnicity**				
White non-Hispanic	7,755	25.0 (23.0–27.1)	69,785	10.2 (9.7–10.7)
Black non-Hispanic	784	23.7 (17.4–31.5)	7,946	11.2 (9.6–13.1)
Hispanic	234	36.1 (26.3–47.3)	3,213	14.0 (11.9–16.3)
Other	739	26.4 (18.1–36.7)	6,408	13.6 (10.8–17.2)
**Education**				
<High school	1,689	28.9 (24.5–33.7)	7,798	16.7 (14.7–18.9)
High school or equivalent	3,472	24.8 (21.7–28.3)	26,858	11.5 (10.6–12.5)
>High school	4,336	25.0 (22.2–28.0)	52,531	9.3 (8.7–9.8)
**Number of adults in household**				
1	4,530	28.8 (26.0–31.8)	32,034	12.3 (11.6–13.1)
2 or more	4,730	24.4 (21.9–27.1)	53,346	10.6 (10.0–11.3)
**Have health insurance**				
No	836	31.4 (24.1–39.8)	7,182	12.8 (11.0–14.7)
Yes	8,663	25.1 (23.1–27.2)	80,024	10.8 (10.3–11.4)
**Smoking status**				
Never smoker	2,213	24.3 (20.5–28.6)	47,077	9.2 (8.5–9.9)
Former smoker	4,256	22.3 (19.8–25.1)	28,947	12.2 (11.3–13.1)
Current smoker	3,016	31.5 (27.8–35.4)	10,913	15.2 (13.6–17.0)
**Physical activity status**				
Any physical activity	5,087	24.7 (22.1–27.6)	62,759	9.7 (9.1–10.3)
No physical activity	4,401	27.1 (24.3–30.0)	24,454	14.6 (13.5–15.7)
**Body mass index (kg/m2)**				
Underweight (<18.5)	314	21.9^c^ (12.2–36.1)	1,224	17.1 (11.1–25.4)
Normal weight (18.5-24.9)	2,611	23.6 (20.3–27.3)	27,199	9.9 (9.1–10.8)
Overweight (25.0-29.9)	2,896	25.9 (22.4–29.7)	31,727	10.2 (9.3–11.2)
Obese (≥30.0)	3,387	28.2 (24.9–31.7)	23,927	13.2 (12.2–14.3)
**High blood pressure**				
No	3,268	22.6 (19.7–25.9)	43,915	9.1 (8.4–9.8)
Yes	6,223	27.5 (25.1–30.1)	43,221	13.2 (12.5–14.0)
**High cholesterol**				
No	3,258	21.4 (18.4–24.6)	40,959	8.5 (7.8–9.2)
Yes	5,593	27.9 (25.3–30.7)	40,211	13.3 (12.5–14.1)
**Heart disease**				
No	6,759	23.3 (21.2–25.6)	77,068	10.2 (9.7–10.8)
Yes	2,728	32.3 (28.3–36.5)	9,215	18.4 (16.5–20.4)
**Stroke**				
No	8,306	23.9 (21.9–26.1)	82,854	10.3 (9.7–10.8)
Yes	1,144	40.1 (34.6–45.8)	4,264	27.1 (23.8–30.6)
**Diabetes**				
No	7,113	24.7 (22.5–27.0)	73,568	10.3 (9.7–10.9)
Yes	2,383	29.2 (25.2–33.5)	13,665	15.1 (13.8–16.5)
**Current asthma**				
No	6,084	23.7 (21.4–26.2)	82,192	10.5 (10.0–11.1)
Yes	3,256	28.5 (25.2–32.1)	4,795	18.3 (15.4–21.6)
**Any chronic health condition^d^**				
No	3,460	21.1 (18.3–24.2)	62,033	9.1 (8.6–9.8)
Yes	6,052	28.4 (25.9–31.1)	25,319	15.8 (14.8–16.9)
**Frequent mental distress**				
No	7,077	18.8 (16.9–20.9)	78,366	8.4 (8.0–8.9)
Yes	2,185	44.1 (39.4–48.9)	7,515	33.2 (30.5–36.0)

Abbreviations: CI, confidence interval; COPD, chronic obstructive pulmonary disease.

a Arkansas, California, Florida, Hawaii, Illinois, Iowa, Louisiana, Maryland, Michigan, Nebraska, New Hampshire, New York, North Carolina, Oklahoma, South Carolina, Tennessee, Texas, Utah, Washington, West Virginia, and Wisconsin.

b Percentages are weighted to state population estimates. Totals may vary because of missing responses.

c Relative standard error is 20% to 30%. Estimate should be used with caution.

d Any chronic health condition: heart disease (heart attack, coronary heart disease), stroke, diabetes, current asthma.

We used logistic regression to further examine the inverse association of age and ICML among people with COPD. When age was examined as a continuous variable, the inverse association of age with ICML among people with COPD was significant (odds ratio [OR]: 0.98; 95% CI: 0.97–0.99, *P* < .001). However, this association was not significant after we adjusted for frequent mental distress (OR: 0.99; 95% CI: 0.98–1.00, *P* = .19). Similar patterns were observed when we examined age grouped into 5-year intervals.

In multivariable analyses (N = 82,013 for all models; 7,888 people with COPD and 74,125 people without COPD) adjusting for demographics, the prevalence ratio of ICML for people with COPD versus those without COPD was 2.25 (95% CI, 2.03–2.49). When further adjusted for health risk behaviors and conditions, the prevalence ratio was 1.69 (95% CI, 1.51–1.89). After additional adjustment for frequent mental distress, the prevalence ratio was 1.48 (95% CI, 1.32–1.66).

The Figure shows 1) general limitations among people with and people without ICML and 2) ICML-associated limitations among those reporting ICML, by COPD status. General limitations among those either with ICML or without ICML were greater among those with COPD than without the condition (*P* < .05). People with both ICML and COPD were more likely than those with ICML but without COPD to report having ICML-associated limitations or having received assistance because of ICML. ICML-associated limitations among those with COPD ranged from almost one-third reporting having received assistance from family or friends because of ICML to almost two-thirds reporting needing assistance in at least 1 of 5 domains (safety, transportation, household activities, personal care, or some other domain).

**Figure:**
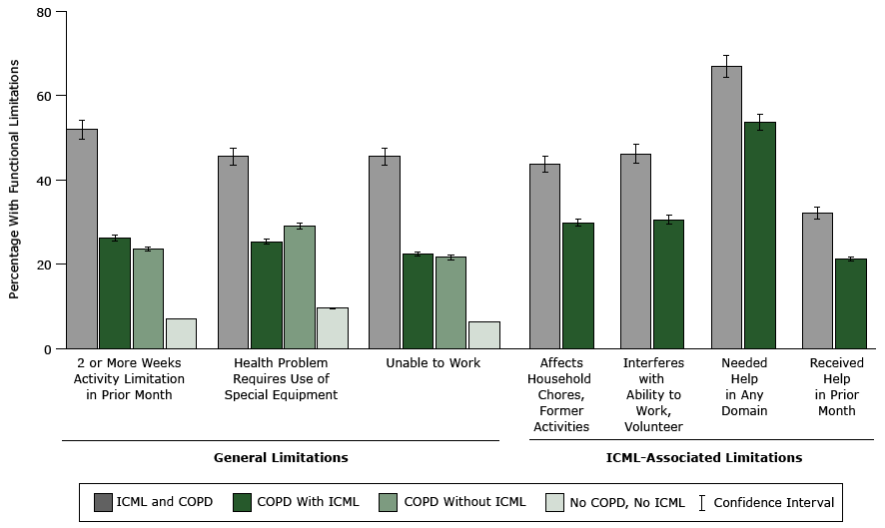
Functional limitations among persons with or without reported increased confusion or memory loss (ICML), by chronic obstructive pulmonary COPD status, adults aged 45 years and older in 21 states (Arkansas, California, Florida, Hawaii, Illinois, Iowa, Louisiana, Maryland, Michigan, Nebraska, New Hampshire, New York, North Carolina, Oklahoma, South Carolina, Tennessee, Texas, Utah, Washington, West Virginia, and Wisconsin), 2011 Behavioral Risk Factor Surveillance System. Domains where help was needed are safety, transportation, household activities, personal care, and other. Abbreviations: CI, confidence interval; COPD, chronic obstructive pulmonary disease; —, not applicable. **Table:** 

Limitation	ICML With COPD, % (95% CI)	ICML Without COPD, % (95% CI)	NO ICML With COPD, % (95% CI)	No ICML or COPD, % (95% CI)
General limitation				
2 or more weeks of activity limitation in prior month	51.9 (47.4-56.3)	26.2 (24.0-28.6)	23.6 (21.5-26.0)	7.0 (6.5-7.4)
Health problem requires use of special equipment	45.5 (41.2-49.9)	25.3 (23.1-27.6)	29.1 (26.6-31.7)	9.5 (9.0-9.9)
Unable to work	45.6 (41.2-50.0)	22.3 (20.1-24.7)	21.6 (19.1-24.2)	6.3 (5.9-6.8)
ICML-associated limitation				
Difficulty with household chores, former activities, or social activities	43.7 (39.3-48.2)	29.8 (27.1-32.5)	—	—
Interferes with ability to work or volunteer	46.2 (41.7-50.6)	30.4 (28.1-32.9)	—	—
Needed help in any domain	66.9 (62.6-70.8)	53.7 (51.1-56.3)	—	—
Received help in prior month	32.1 (28.1-36.4)	21.2 (19.0-23.5)	—	—

When adjusted for all covariables (Table 3), people with COPD remained more likely than those without COPD to report each general activity limitation. This was true for both those with and those without ICML. For ICML-related limitations, after full adjustment, people with ICML and COPD were more likely than those with ICML but no COPD to report interference with work, volunteering, or social activities (aPR,1.17; 95% CI, 1.01–1.36) because of ICML.

**Table 3:** Multivariable Adjusted Associations of Functional Limitations Among Adults Aged 45 Years or Older With Versus Without COPD, By ICML Status, 21 States^a^, 2011 Behavioral Risk Factor Surveillance System

Limitation	N	With ICML, PR (95% CI)^b^			N	Without ICML, PR (95% CI)^b^		
		Model 1^c^	Model 2^d^	Model 3^e^		Model 1^c^	Model 2^d^	Model 3^e^
**General limitation**								
Activity limited for 2 weeks or more in prior month due to physical or mental health	8,645	1.85 (1.63–2.10)	1.54 (1.35–1.77)	1.43 (1.26–1.63)	72,695	3.36 (2.96–3.81)	2.16 (1.88–2.49)	1.82 (1.59–2.09)
Health problem required use of special equipment, such as a cane, wheelchair, special bed, or special telephone	8,798	1.65 (1.42–1.91)	1.34 (1.16–1.57)	1.33 (1.14–1.54)	73,165	2.53 (2.27–2.82)	1.83 (1.64–2.05)	1.74 (1.55–1.95)
Unable to work	8,788	1.92 (1.66–2.21)	1.46 (1.24–1.72)	1.42 (1.20–1.67)	73,071	3.40 (2.92–3.97)	2.10 (1.76–2.50)	1.92 (1.60–2.30)
**ICML related limitation**								
Gave up household chores or former activities due to ICML	8,589	1.44 (1.25–1.66)	1.19 (1.02–1.38)	1.15 (0.98–1.34)	—	—	—	—
ICML interfered with ability to work, volunteer, or engage in social activities	8,577	1.45 (1.27–1.66)	1.22 (1.05–1.41)	1.17 (1.01–1.36)	—	—	—	—
Needed assistance in a domain^f^	8,446	1.23 (1.14–1.34)	1.11 (1.01–1.21)	1.09 (1.00–1.19)	—	—	—	—
Family or friends provided assistance in prior 30 days due to ICML	8,613	1.33 (1.10–1.60)	1.10 (0.89–1.35)	1.06 (0.87–1.30)	—	—	—	—

Abbreviations: CI, confidence interval; COPD, chronic obstructive pulmonary disease; ICML, increased confusion or memory loss; PR, prevalence ratio; —, not applicable.

a Arkansas, California, Florida, Hawaii, Illinois, Iowa, Louisiana, Maryland, Michigan, Nebraska, New Hampshire, New York, North Carolina, Oklahoma, South Carolina, Tennessee, Texas, Utah, Washington, West Virginia, and Wisconsin.

b Comparison is between people with COPD and people without COPD (referent group).

c Adjusted for sex, race, education, state, and age.

d Adjusted for model 1 plus risk behaviors/factors and at least 1 other chronic condition (heart disease, stroke, diabetes, asthma).

e Adjusted for models 1 and 2 plus frequent mental distress (2 weeks or more when mental health was not good).

f Domains are safety, transportation, household activities, personal care, and other.

## Discussion

Associations between COPD and cognitive impairment are complex and may be due to numerous factors, including hypoxia or hypercapnia, combined effects of co-occurring chronic conditions with COPD, or effects of tobacco smoking. Although we did not have information on severity of either COPD or ICML, our results in a large sample of community dwelling adults aged 45 years or older suggested that people with COPD were more likely than those without COPD to report confusion or memory loss that worsened over the previous year, even after controlling for numerous potential confounders, such as demographic variables, co-occurring health risk factors and conditions, and frequent mental distress. General limitations were greater among those with COPD than those without regardless of ICML status, and the percentages reporting ICML-associated difficulties were substantial in this community-dwelling sample.

Subjective cognitive decline has received considerable attention in the literature as interest has grown in identifying early markers of cognitive decline. Previous studies suggest that subjective memory complaint is associated with psychological distress, yet is also independently related to development of dementia (8–11). We observed a higher prevalence of frequent mental distress among people with COPD than among those without, and associations between COPD and ICML were attenuated but remained significant after adjustment for frequent mental distress, suggesting that both ICML and psychological distress are associated with COPD. Both psychological distress and cognitive functioning can affect treatment, self-management, and quality of life for people with COPD; therefore, consideration of both factors may be important. A review of a growing body of research suggests that specific behavioral and psychological aspects of dementia might be amenable to intervention (28). Psychological factors involved with COPD may be addressed by pulmonary rehabilitation, although the benefits of pulmonary rehabilitation for cognitive decline among people with COPD remains unclear (29).

Our observation of an inverse association of ICML with age among people with COPD was at least partly explained by frequent mental distress, confirming potential confounding between psychological distress and ICML (20–24). Several other reasons should also be considered for results based on surveys of the general population. First, it is possible that recognition of memory problems is greater when symptoms and their effects on functional status are first noticed, especially if attributed to another condition. Additionally, our sample consisted of community-dwelling adults; adults with COPD or ICML who reside in institutional settings (who may have more severe COPD or ICML) are not represented. In a study that included 27,106 people with COPD in US nursing homes from 2009 through 2010, 61.9% had short-term memory problems and 43.3% had moderately or severely impaired cognitive skills (30). Finally, although the association of ICML and age by COPD status may be confounded by psychological distress, the inverse association of ICML with age among people with COPD may signify a healthy survivor effect.

Several limitations should be noted (25,26). First, these analyses relied on self-report of both COPD and ICML. How cognitive impairment influences self-reports is unclear. For example, respondents need to be able to answer questions over the telephone for the BRFSS, and it is not known how ICML might affect responses or ability to report functional limitations. BRFSS questions were cognitively tested but were not validated against a clinical population or measurement for either condition, although the COPD question is structured as in other health surveys. Self-reported information is also subject to recall and social desirability biases. Additionally, although we had a large sample, these results were based on adults in 21 states, limiting generalizability. Finally, analyses were cross-sectional and causality cannot be inferred. Furthermore, although we controlled for numerous chronic conditions, we were not able to control for neurological conditions other than stroke. However, our results support prior observations that those with COPD have more self-reported memory complaints. Also, because cognitive problems may begin many years before other symptoms appear or a clinical diagnosis is made, self-reports or those of proxy respondents are important to consider.

Although further research is needed, our results can inform treatment and self-management of people living with COPD. Because even middle-aged adults (age 45–64 y) with COPD were more likely to report ICML than were middle-aged people without COPD, health care providers should consider assessing the cognitive functioning of their patients with COPD because impairments may affect treatment and management outcomes. Furthermore, although the associations between ICML and COPD status was attenuated with adjustment for frequent mental distress, these associations remained, suggesting that both cognitive impairment and psychological distress should be assessed and monitored for effective treatment and self-management. Finally, specific functional limitations that may be of particular concern to people with COPD can be addressed.
